# Cardiovascular Disease and Hair Cortisol: a Novel Biomarker of Chronic Stress

**DOI:** 10.1007/s11886-019-1208-7

**Published:** 2019-08-30

**Authors:** Eleonora Iob, Andrew Steptoe

**Affiliations:** Department of Behavioural Science and Health, University College London, UCL, Gower Street, London, WC1E 6BT UK

**Keywords:** Chronic stress, Hair cortisol, Cardiovascular disease, Cardiometabolic markers

## Abstract

**Purpose of Review:**

This review focuses on the concentration of cortisol in human hair as a biomarker of chronic stress in cardiovascular disease (CVD). We outline the cardiovascular consequences of cortisol excess and provide a comprehensive overview of recent studies investigating the relationship of hair cortisol with CVD. In addition, clinical implications and limitations of the evidence are discussed, together with directions for future research.

**Recent Findings:**

Hair cortisol may be a reliable biomarker of chronic stress since it provides quantification of total cortisol secreted into hair over several weeks. A growing body of evidence suggests that elevated hair cortisol levels are associated with both the incidence of CVD and poorer recovery and treatment outcomes. Moreover, increased hair cortisol concentration has been linked with established cardiometabolic risk factors for CVD including high blood pressure, diabetes, and adiposity.

**Summary:**

Hair cortisol is a promising biomarker of chronic cortisol excess which may contribute to both the pathogenesis and prognosis of CVD. However, the current evidence relies on small-scale cross-sectional studies. Further research adopting longitudinal designs across larger samples of CVD patients and healthy participants is required to inform the development of novel evidence-based interventions.

## Introduction

Cardiovascular disease (CVD) is a leading contributor to the burden of morbidity and mortality across the world. Although the prevalence of CVD has declined significantly over the last two decades in many countries [1], cardiovascular conditions such as coronary heart disease and stroke remain the two most common causes of disease worldwide according to Global Burden of Disease and World Health Organization estimates [2, 3]. Moreover, the levels of CVD-related deaths and years lost due to ill health or premature death have increased considerably since 2006 owing to population growth and ageing. Hence, the identification of modifiable risk factors and novel targets for preventive interventions is an issue of major public health concern.

Established cardiometabolic risk factors which are known to substantially increase CVD risk include elevated cholesterol levels [4], high blood pressure [5], excessive weight gain and obesity [6] and diabetes [7]. Whilst strong effects have been found for cigarette use [8], the contribution of other behavioural factors such as physical inactivity and alcohol consumption appears less consistent [9, 10]. Recently, greater attention has been devoted to the role of psychosocial stress in CVD as a result of increasing knowledge of its adverse physiological consequences for both mental and physical health [11]. A significant body of evidence indicates that acute and chronic stressors (e.g. childhood trauma, work stress, social isolation) and negative emotional states (e.g. depression, anxiety) can influence the development of CVD and triggering of cardiovascular events independently of classical CVD risk factors [12–15]. The elevated CVD risk associated with a history of multiple stressful events during childhood is comparable to that observed for several cardiometabolic and behavioural risks [12]. Adult stress appears to have a more influential role in the triggering of cardiovascular events rather than in the aetiology of the disease [16••]. Psychosocial stress also acts as a prognostic factor contributing to the course, progression, and outcomes of CVD [17–20]. Additionally, conventional risk factors for CVD including hypertension [21], diabetes [22], adiposity [23], smoking [24], and physical inactivity [25] are negatively affected by stress.

The relationship between psychosocial stress and CVD is now well documented in the epidemiological and clinical research literature. Yet, the psychobiological processes through which stress contributes to the pathogenesis and prognosis of CVD remain elusive. Dysregulation of the hypothalamic-pituitary-adrenal (HPA)-axis has been proposed to underlie the adverse physiological effects of stress on CVD [16••]. Traditionally, HPA-axis function has been measured through the assessment of cortisol levels in saliva, blood, or urine. However, the concentration of cortisol secreted into hair has recently emerged as a novel biomarker of long-term HPA-axis activity offering several advantages over traditional specimens [26]. This review focuses on the role of hair cortisol as a biomarker of chronic stress in CVD. First, the cardiovascular effects of chronic cortisol exposure are described. Second, we discuss the main limitations of cortisol measurements in body fluids (i.e. saliva, blood, and urine) and outline key advantages of cortisol quantification in human hair. Third, we provide a comprehensive overview of emerging studies investigating the association of hair cortisol with CVD incidence, prognosis and cardiometabolic risk factors. Lastly, clinical applications, limitations of the current evidence and directions for future research are discussed.

## The Role of Chronic Exposure to Cortisol in CVD

### Physiology of the Stress Response

**Fig. 1:**
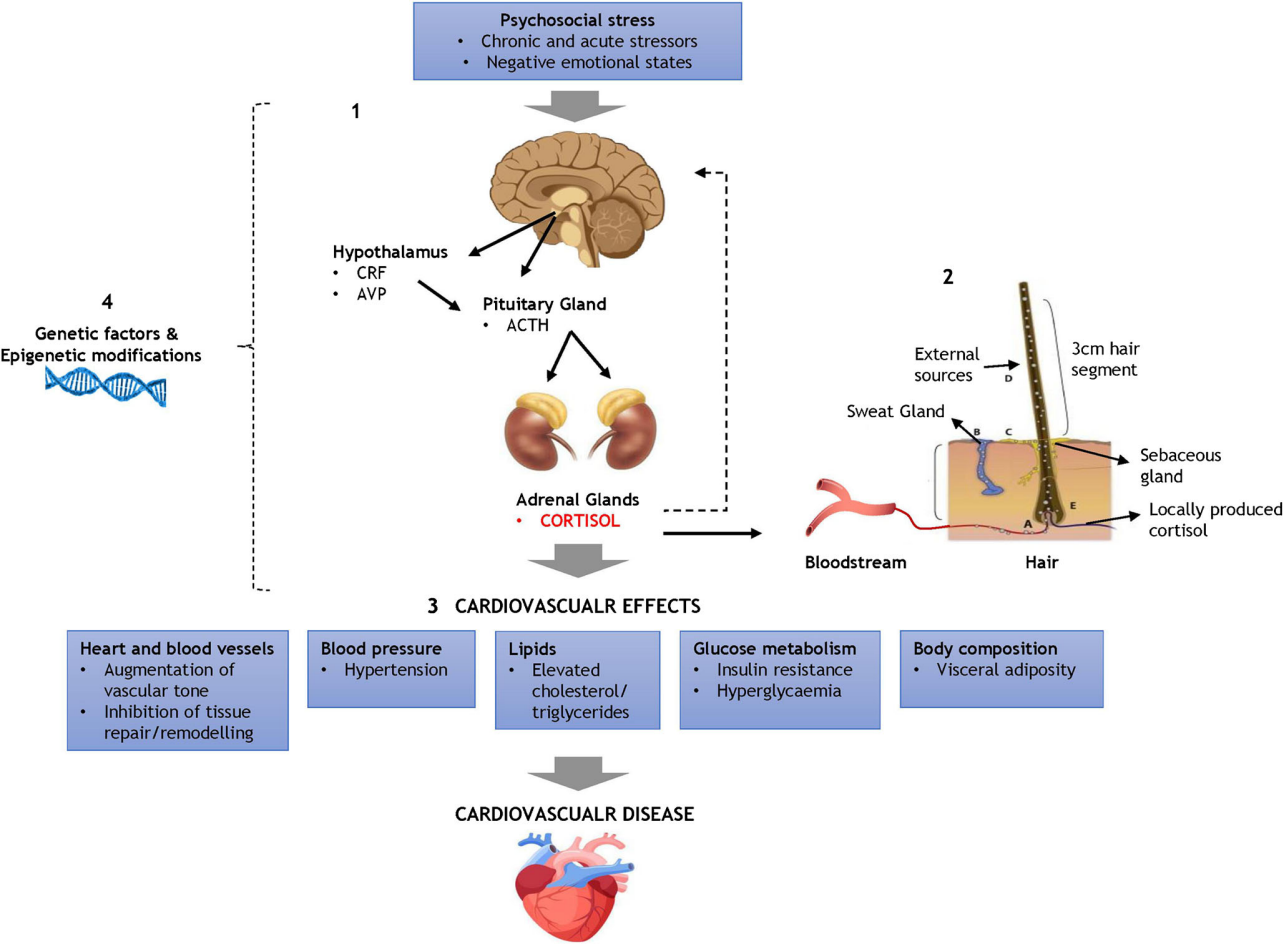
Mechanisms through which psychosocial stress leads to elevated hair cortisol concentration and affects cardiovascular disease (CVD) risk and prognosis. 1 HPA-axis response to psychosocial stress. 2 Incorporation of cortisol into hair. 3 Cardiovascular effects of cortisol. 4 Role of genetic factors and epigenetic processes. (Hair image adapted from Stalder & Kirschbaum, 2012) [26]

The HPA-axis is a crucial stress response system in humans. Its primary function is to maintain homeostasis and facilitate successful adaptation to the surrounding environment [27]. This is achieved through a cascade of hormonal reactions involving the hypothalamus, the pituitary gland and the adrenal cortex [28] (Fig. 1). The activation of the stress response begins with the release of corticotropin releasing factor (CRF) and vasopressin (AVP) from the hypothalamus. Increased levels of these hormones stimulate the production of adrenocorticotropic hormone (ACTH) from the pituitary gland. This in turn prompts the adrenal gland to release glucocorticoids including cortisol, the primary end-product of the HPA-axis [28]. Glucocorticoids are transported into the blood stream by corticosteroid binding globulin (CBG) and readily diffuse through cellular membranes by binding to both mineralocorticoid receptors (MR) and glucocorticoid receptors (GR) [29].

Acute and transient HPA-axis activation has an adaptive function since it facilitates effective coping with external stressors by triggering a number of physiological reactions. These include, for instance, increased vascular tone, immune activation, suppression of inflammation, energy mobilisation, insulin resistance, inhibition of reproductive physiology and behaviour and sharpened cognition [30]. However, prolonged and repeated cortisol exposure is maladaptive and may lead to long-term physiological alterations compromising the function of the cardiovascular, metabolic, immune and nervous systems [11]. Consequently, chronic stress may increase the individual’s susceptibility to poor health and disease.

### Cardiovascular Consequences of Chronic Cortisol Excess

The unfavourable effects of chronic cortisol excess on the body and the brain are particularly relevant for the pathogenesis and progression of CVD. Details of the impact of chronic cortisol excess on the development of CVD are beyond the scope of this review, but include hyperlipidaemia, insulin resistance, hyperglycaemia, hypertension and abdominal adiposity [29, 31] (Fig. 1). Briefly, elevated glucocorticoid output can affect plasma lipoprotein metabolism resulting in elevated levels of cholesterol and triglycerides. Cortisol opposes the action of insulin and activate gluconeogenesis in the liver thereby contributing to insulin resistance and hyperglycaemia [29]. Elevated glucocorticoids are known to have harmful effects on blood pressure and lead to hypertension. Candidate mechanisms in the development of cortisol-induced hypertension include mineralocorticoid-induced sodium retention, plasma volume expansion, and inhibition of vasodilator hormones [31]. Moreover, increased glucocorticoid output is linked with a number of metabolic changes, such as greater adipocyte development and increased 11β-hydroxysteroid dehydrogenase I (HSD11B1) activity in adipocytes, which can augment visceral adiposity and risk of abdominal obesity. In turn, increased adiposity contributes to other CVD risk factors since fat cells release hormones and metabolites that adversely affect blood pressure, plasma lipoproteins, coagulation and insulin resistance [29].

Cortisol has a direct impact on the heart and blood vessels as well as systemic effects on cardiometabolic markers [32]. GR and MR are expressed in the circulatory system where glucocorticoids are involved in the maintenance of vascular tone and in the modulation of inflammatory, proliferative and remodelling responses to injury and vascular occlusion [33]. Such local effects have been proposed to play an important role not only in the process of atherogenesis but also in the progression of CVD [32].

Compelling evidence for the adverse cardiovascular consequences of chronic cortisol excess has been provided by studies of patients with endogenous hypercortisolism and those treated with glucocorticoid therapy. For instance, patients with Cushing’s syndrome, a condition characterised by chronic cortisol excess, are estimated to have a fourfold higher risk of CVD mortality, higher rates of CVD complications, and greater incidence of cardiometabolic risk factors (e.g. obesity, hypertension, insulin resistance) compared to healthy controls [31, 34, 35]. Likewise, patients treated with high doses of glucocorticoids have been shown to have substantially higher risk of cardiovascular events and adverse cardiometabolic markers [32, 36].

## Methods for Assessing Cortisol Levels

### Measurement of Cortisol in Blood, Urine, and Saliva

Given the elevated CVD risk observed in patients with Cushing’s syndrome and those treated with glucocorticoid therapy, several studies have investigated the relationship of endogenous cortisol levels with the development and progression of CVD in both clinical and population-based samples. Endogenous cortisol levels have traditionally been assessed through measurements in body fluids including saliva, blood and urine [37]. Blood samples provide measures of circulating levels of both CBG-bound and bioactive (free) cortisol at a single time point. In contrast, urinary measures of cortisol reflect the total exposure to bioactive cortisol across 12 or 24 h [38]. Bioactive cortisol levels can also be measured via saliva samples. However, as for blood samples, salivary cortisol only provides quantification of cortisol concentration at a single time point [37]. Since cortisol has a marked diurnal rhythm, multiple blood or saliva samples over the day are needed to characterise the overall profile of cortisol secretion. Repeated blood sampling is difficult to carry out except in clinical or laboratory settings, so saliva has been increasingly favoured as less obtrusive. These methods are particularly useful in acute laboratory stress paradigms for monitoring acute HPA-axis responses [39].

Overall, this work has provided evidence that cortisol dysregulation is associated with CVD incidence [40–44], prognosis [45, 46] and mortality [47, 48], as well as with cardiometabolic risk factors such as hypertension, hyperlipidaemia, hyperglycaemia and diabetes [49–56]. However, not all studies have found evidence supporting this link [49, 57–62], and some have reported reduced HPA-axis activity [63–66]. Additionally, there is little agreement on the types of cortisol measures that might be more predictive of CVD risk and progression across studies.

These inconsistent results could be explained by the limitations of cortisol quantification in body fluids. A fundamental issue with these methods is that they only provide momentary or short-term estimates of cortisol levels, rather than information on long-term exposure to elevated cortisol [26]. Cortisol concentration in saliva, blood and urine is also subject to several situational and interindividual fluctuations due to various confounding factors such as circadian rhythm, momentary moods and events and study procedures. Few studies take samples over several days, and the days selected may not be representative. The collection of repeated cortisol measures over the course of a day can lead to excessive participant burden and incomplete sample collection [67]. Crucially, prolonged and repeated exposure to elevated cortisol levels is likely to play a more important role in the aetiology and progression of CVD than acute and short-term cortisol reactivity. Indeed, acute and transient cortisol exposure is typically unharmful and promotes successful adaptation to the environment. In contrast, chronically elevated cortisol levels are maladaptive and related to ill health [68••].

### Hair Cortisol: a Novel Biomarker of Chronic Cortisol Exposure

Since the discovery of glucocorticoids in hair in 2004 [69], several lines of research have suggested that hair cortisol is a valid biomarker of psychosocial stress in both children and adults [67, 70–73]. Quantification of cortisol in hair offers several advantages over traditional specimens. In particular, hair cortisol may be a reliable biomarker of long-term HPA-axis activity since it reflects total cortisol output over several weeks or months. Hair analysis also offers a non-invasive, low-burden and single-sample measurement of HPA-axis activity which does not rely on participant adherence to collection instructions. Additionally, hair cortisol concentration is less influenced by situational and interindividual variations than traditional methods and has high test-retest reliability [26, 38]. Hence, hair cortisol may prove to be a suitable biomarker for studying the cardiovascular consequences of chronic stress in both clinical settings and large-scale population studies.

Incorporation of cortisol into hair may occur through a number of mechanisms (Fig. 1). It has been suggested that the free cortisol present in follicular capillaries is incorporated into the medulla of the hair shaft via passive diffusion during hair growth. Thus, the amount of cortisol deposited into hair is likely to reflect biologically active hormone [38], and to be proportional to the concentration of systemic cortisol [74]. It has also been proposed that cortisol is deposited onto the hair shaft through sweat and sebaceous glandular secretions [69]. Additionally, a local HPA-like pathway in the hair follicles has been suggested [75]. The extent to which these different sources contribute to hair cortisol content remains unclear.

While there are some individual and ethnic variations, human hair is generally estimated to grow approximately 1 cm per month [76]. Segmental hair analysis therefore provides the opportunity to use cortisol content in each centimetre of hair as a proxy measure of HPA-axis activity during the month represented by the respective hair segment [67]. Unfortunately, cortisol can only be reliably estimated in the 6 cm closest to the scalp because more distal hair segments contain lower hormone levels due to greater environmental damage [76]. Concentrations of cortisol in hair are affected by repeated shampooing, chemical treatments (e.g. demi-perms, bleach) and sun exposure, so these factors need to be taken into account [77••]. The posterior vertex has been shown to have less variation in cortisol levels than other areas of the scalp [78]. Consequently, most studies typically collect a 3-cm segment of hair from the posterior vertex as close to the scalp as possible in order to represent total cortisol output over the preceding 3 months [67]. After collection, hair samples are typically weighted, washed and either minced or grounded. Cortisol is then extracted from minced or ground hair using organic solvents. Finally, the extracted cortisol is quantified using an immunoassay or liquid chromatography tandem–mass spectrometry (LC–MS/MS) [74]. Although their agreement in terms of absolute values is low, correlations between cortisol levels determined by these two methods are generally high [79]. It should be noted that the absolute concentration of cortisol is very different in hair, blood, saliva and urine, so each system has its own distribution and range.

## Research Evidence for the Relationship of Hair Cortisol with CVD

### Association of Hair Cortisol with CVD Incidence and Prognosis

A systematic search of all published studies up to May 2019 using PubMed, Ovid Medline and Web of Science revealed 11 studies examining the association of hair cortisol with the incidence or recovery from CVD (Table 1). Four case-control studies found evidence for higher hair cortisol levels in patients with acute coronary syndrome [80•], myocardial infarction [81•], coronary heart disease [83•] and aneurysmal subarachnoid haemorrhage [82•] compared with control participants. In addition, a population-based study revealed that higher hair cortisol was associated with an increased incidence of coronary heart disease, stroke and peripheral arterial disease [84•]. In contrast, coronary heart disease diagnosis or the experience of a stroke were unrelated to hair cortisol in a large observational cohort [85•]. However, hair cortisol was positively associated with other CVD risk factors (i.e. BMI, diabetes) and CVD medication in this study, and the authors suggest that hair cortisol might be more predictive of CVD risk rather than being an actual marker of CVD.

**Table 1:** Association of hair cortisol with the incidence and prognosis of CVD

Factors		Significant association	Sample Size	Duration	Refs
CVD incidence					
Condition					
Acute coronary syndrome		Yes	166	Cross-sectional	[80•]
Acute myocardial infarction		Yes	112	Cross-sectional	[81•]
Aneurysmal subarachnoid haemorrhage		Yes	32	Cross-sectional	[82•]
Coronary heart disease		Yes	598	Cross-sectional	[83•]
Coronary heart disease/stroke/peripheral arterial disease		Yes	283	Cross-sectional	[84•]
Coronary heart disease/stroke		No	3675	Cross-sectional	[85•]
CVD prognosis					
Condition	*Outcome/predictor*				
Chronic heart failure	Symptom severity	Yes	44	Prospective	[86•]
Coronary artery disease	Recovery	Yes	56	Prospective	[87•]
Stroke	Recovery	Yes	65	Prospective	[88•]
Acute coronary syndrome	Psychological distress	No	121	Cross-sectional	[89]
Aneurysmal subarachnoid haemorrhage	Psychological distress	Yes	32	Cross-sectional	[82•]
Structural heart disease	Psychological distress	Yes	261	Prospective	[90•]
Structural heart disease	Physical health status	Yes	261	Cross-sectional	[90•]

Hair cortisol concentration has also been investigated as a prognostic factor in CVD. One study revealed a positive relationship between hair cortisol and the severity of symptoms in a sample of patients with chronic heart failure [86•]. Over a 1-year follow-up, there also was a positive albeit non-significant trend towards higher hair cortisol in patients who had CVD-related hospitalisations compared with non-hospitalised patients. Elevated hair cortisol levels predicted poorer memory improvement in a sample of patients with coronary artery disease attending a 1-year cardiac rehabilitation intervention [87•], while another study demonstrated that higher hair cortisol concentration was associated with larger lesion volume and worse cognitive results 6, 12 and 24 months following stroke [88•]. Elevated hair cortisol has also been associated with greater psychological distress in patients with aneurysmal subarachnoid haemorrhage [82•]. Another study found that higher hair cortisol concentration was related to worse subjective physical health status in patients with structural heart disease (cardiomyopathy, congenital heart disease or coronary heart disease), while a more favourable mental health status predicted a decline in cortisol levels at 12-week follow-up [90•]. By contrast, another study of patients with acute coronary syndrome found no evidence supporting the link between hair cortisol and depressive symptoms [89].

### Association of Hair Cortisol with Cardiometabolic Risk Factors

A larger number of studies have investigated the relationship of hair cortisol with cardiometabolic risk factors. Table 2 provides an overview of the evidence available to date. A meta-analysis of 11 studies has corroborated the positive association of hair cortisol with systolic blood pressure, whereas the relationship with diastolic blood pressure was overall non-significant [68••]. Elevated hair cortisol levels have also been related to adverse metabolic blood markers such as cholesterol, triglycerides and glycated haemoglobin [91–93, 95], although associations were not significant in all cases [94]. Thus, the relationship of hair cortisol with lipids varied considerably across studies. Two studies calculated a composite score of cardiometabolic risk based on the diagnostic criteria for the metabolic syndrome which was found to be positively associated with hair cortisol [91, 93]. Moreover, virtually all studies to date have confirmed the presence of elevated hair cortisol levels in people with diabetes [84, 85, 96–98]. Robust effects have also been reported for the link between hair cortisol and adiposity, and a meta-analysis confirmed the association with higher body mass index (BMI) and waist-hip ratio [68]. Interestingly, there is evidence suggesting that hair cortisone is linked with unfavourable cardiometabolic markers, and these associations are sometimes stronger than those for cortisol [91, 93, 94]. Cortisone is another glucocorticoid hormone which is directly metabolised from cortisol by HSD11B enzymes. It is generally considered to be an inactive metabolite since it has considerably lower glucocorticoid activity than cortisol [69]. Therefore, the assessment of hair cortisone along with cortisol could provide greater insight into long-term levels of both active and inactive glucocorticoids in the body [77••]. Overall, despite the presence of some inconsistent findings, current research provides intriguing evidence for the adverse cardiometabolic effects of chronic cortisol excess as assessed in human hair.

**Table 2:** Association of hair cortisol with cardiometabolic risk factors for CVD

Risk factors	Significant association	Sample Size	Duration	Ref
Diastolic blood pressure	No	2832^b^	Cross-sectional	[68••]
Systolic blood pressure	Yes	2832^b^	Cross-sectional	[68••]
Cholesterol	Yes^a^	1258	Cross-sectional	[91]
Cholesterol	Yes	163	Cross-sectional	[92]
Cholesterol	No	85	Cross-sectional	[93]
Cholesterol	No	295	Cross-sectional	[94]
Triglycerides	No	163	Cross-sectional	[92]
Triglycerides	Yes	85	Cross-sectional	[93]
Triglycerides	No	1258	Cross-sectional	[91]
Triglycerides	No	295	Cross-sectional	[94]
Glucose	No	1258	Cross-sectional	[91]
Glucose	No	85	Cross-sectional	[93]
Glucose	No	295	Cross-sectional	[94]
Glycated haemoglobin	Yes	1258	Cross-sectional	[91]
Glycated haemoglobin	Yes	61	Cross-sectional	[95]
Glycated haemoglobin	No	295	Cross-sectional	[94]
Metabolic syndrome	Yes	1258	Cross-sectional	[91]
Metabolic syndrome	Yes	85	Cross-sectional	[93]
Diabetes	Yes	3675	Cross-sectional	[85]
Diabetes	Yes	760	Cross-sectional	[96]
Diabetes	Yes	654	Cross-sectional	[97]
Diabetes	Yes	283	Cross-sectional	[84•]
Diabetes	Yes	55	Cross-sectional	[98]
Waist-hip ratio	Yes	3202^b^	Cross-sectional	[68••]
Body mass index	Yes	8062^b^	Cross-sectional	[68••]

^a^Negative association

^b^Meta-analysis

## Clinical Applications

Hair cortisol may be valuable not only in understanding the development and prognosis of CVD, but also in prevention and treatment. However, the impact of psychosocial, behavioural and pharmacological interventions on long-term cortisol levels is not yet known. The potential benefits for CVD of psychosocial treatment focusing on stress management have been demonstrated in several investigations [16••]. A Cochrane review of 35 studies published in 2017 concluded that psychosocial treatment might lead to reduced cardiovascular mortality among people with coronary heart disease, but noted that many trails have been of low quality and do not provide robust data [99]. On the other hand, studies on the general population indicate that psychosocial interventions such as cognitive behavioural therapy (CBT), mindfulness, yoga or green space exposure are associated with reductions in cortisol levels and other stress-related hormones, as well as with a more favourable cardiometabolic risk profile [100–103]. In addition, it has been suggested that pharmacological therapy reducing glucocorticoid exposure could have a beneficial action on the pathogenesis and progression of CVD [32, 104]. However, there is as yet no direct evidence supporting the efficacy of interventions reducing chronic cortisol levels for the prevention and treatment of CVD.

There is a small number of studies incorporating hair cortisol into intervention studies. For instance, hair analysis has been included in a mindfulness trial for smoking cessation. This study compared the hair segment corresponding to the pre-intervention period to that indexing the post-intervention phase and demonstrated a significant decrease in cortisol concentration after the mindfulness intervention [105]. Another randomised controlled trail of 151 patients with structural heart disease who underwent either mindfulness training or CBT showed a significant decrease in hair cortisol after the intervention in both groups [90•]. These findings highlight the potential of hair cortisol to strengthen the evidence for the effectiveness of different types of stress reduction interventions for CVD.

## Open Questions and Future Directions

The research literature on hair cortisol and CVD is still in its infancy. Although the results are promising, the evidence presented in this review is predominantly based on small-scale cross-sectional studies. Larger prospective studies including repeated assessments of hair cortisol and CVD are required in order to obtain estimates that are more robust and clarify the direction of associations. Furthermore, new intervention studies are needed to test the effectiveness of psychosocial and medical interventions that reduce cortisol levels in the long term. Such work will provide stronger evidence for the possible casual effect of chronic stress on CVD and inform the development of novel preventive and treatment strategies. A number of limitations should be noted. Some people are reluctant to provide hair samples, and baldness or lack of sufficient hair becomes more common with increasing age. Hair assessment is also not suitable for the study of short-term cortisol response to stress or transient clinical events.

This review has only focused on the effect of stress on long-term cortisol levels and CVD. However, elevated cortisol levels could also result from unhealthy behaviours such as smoking [92], excessive alcohol consumption [84•] and physical inactivity [106]. Psychosocial stress may have both a direct effect on CVD and an indirect relationship mediated by health behaviours [107], although studies relating lifestyle factors with hair cortisol have produced inconsistent results [68••, 85•, 96].

Recent developments in genotyping methods and the availability of large-scale genetic consortia will increase our knowledge of the role of genetic and epigenetic factors in the relationship between cortisol and CVD. Certain individuals might be more susceptible to stress-induced CVD owing to their genetic make-up [29, 32]. Genome-wide association studies (GWAS) have documented associations between numerous single nucleotide polymorphisms (SNPs) and CVD [108] and have helped identify genetic variants in biomarkers that play a causal role in aetiology [109]. However, the largest GWAS meta-analysis of plasma cortisol to date has only identified three SNPs that were significantly associated with cortisol concentrations [110]. This result could be explained by the limitations of cortisol measurement in body fluids and the relatively small sample size of the study (*N* = 12,597). Despite low SNP-based heritability, a Mendelian randomisation study has found evidence consistent with a causal effect of plasma cortisol on CVD [111]. A GWAS meta-analysis of hair cortisol is currently underway. This work will help to elucidate the genetic basis of cortisol and provide reliable evidence for drug targets. Other studies have focused on the pathways through which stress may affect epigenetic regulation of individual HPA-axis genes and risk of stress-related disorders including CVD. Such work has demonstrated that DNA methylation of genes implicated in glucocorticoid regulation is linked with hypertension and subclinical atherosclerosis [112]. Future studies should seek to identify epigenome-wide DNA methylation associated with hair cortisol and examine their relationship with CVD.

## Conclusions

The research discussed in this review contributes to the growing body of evidence suggesting that chronically elevated cortisol levels are not only implicated in the aetiology of CVD but also have modulating effects on its progression and treatment. The analysis of hair cortisol offers the opportunity to reliably assess long-term exposure to cortisol and examine its relationship with CVD and cardiometabolic risk factors (Fig. 1). Hair cortisol may be useful in studies of the effects of psychosocial, behavioural or pharmacological treatments on CVD prognosis. Unfortunately, the evidence available to date predominantly relies on relatively small cross-sectional studies, limiting the generalisability and reliability of the findings. Further studies employing longitudinal designs across larger samples of patients with CVD and healthy participants will have the potential to increase our understanding of the cardiovascular consequences of chronic cortisol excess and inform the development of more effective preventive and treatment interventions.
